# The incorporation of focused history in checklist for early recognition and treatment of acute illness and injury

**DOI:** 10.1186/s12873-016-0099-9

**Published:** 2016-08-31

**Authors:** Namita Jayaprakash, Rashid Ali, Rahul Kashyap, Courtney Bennett, Alexander Kogan, Ognjen Gajic

**Affiliations:** Multidisciplinary Epidemiological and Translational Research in Intensive Care, Emergency and Perioperative Medicine (METRIC-EPM), Critical Care Medicine, Mayo Clinic, Mary Brigh building, 2nd floor, 200 1st Street SW, Rochester, MN 55905 USA

## Abstract

**Background:**

Diagnostic error and delay are critical impediments to the safety of critically ill patients. Checklist for early recognition and treatment of acute illness and injury (CERTAIN) has been developed as a tool that facilitates timely and error-free evaluation of critically ill patients. While the focused history is an essential part of the CERTAIN framework, it is not clear how best to choreograph this step in the process of evaluation and treatment of the acutely decompensating patient.

**Methods:**

An un-blinded crossover clinical simulation study was designed in which volunteer critical care clinicians (fellows and attendings) were randomly assigned to start with either obtaining a focused history choreographed in series (after) or in parallel to the primary survey. A focused history was obtained using the standardized SAMPLE model that is incorporated into American College of Trauma Life Support (ATLS) and Pediatric Advanced Life Support (PALS). Clinicians were asked to assess six acutely decompensating patients using pre – determined clinical scenarios (three in series choreography, three in parallel). Once the initial choreography was completed the clinician would crossover to the alternative choreography. The primary outcome was the cognitive burden assessed through the NASA task load index. Secondary outcome was time to completion of a focused history.

**Results:**

A total of 84 simulated cases (42 in parallel, 42 in series) were tested on 14 clinicians. Both the overall cognitive load and time to completion improved with each successive practice scenario, however no difference was observed between the series versus parallel choreographies. The median (IQR) overall NASA TLX task load index for series was 39 (17 – 58) and for parallel 43 (27 – 52), *p* = 0.57. The median (IQR) time to completion of the tasks in series was 125 (112 – 158) seconds and in parallel 122 (108 – 158) seconds, *p* = 0.92.

**Conclusion:**

In this clinical simulation study assessing the incorporation of a focused history into the primary survey of a non-trauma critically ill patient, there was no difference in cognitive burden or time to task completion when using series choreography (after the exam) compared to parallel choreography (concurrent with the primary survey physical exam). However, with repetition of the task both overall task load and time to completion improved in each of the choreographies.

**Electronic supplementary material:**

The online version of this article (doi:10.1186/s12873-016-0099-9) contains supplementary material, which is available to authorized users.

## Background

Diagnostic errors and delayed diagnoses act as a blind spot in the delivery of health care and have been attributed to avoidable illness and death in the United States [1, 2]. Errors in diagnosis occur with common diseases and are not limited to rare conditions [3]. The Institute of medicine’s report on improving diagnosis in healthcare recommends appropriate education and training for healthcare professionals involved in the diagnostic process with use of measures that better incorporate health information technology [1].

The introduction of advanced trauma life support (ATLS), in 1980, standardized trauma care delivered by frontline clinicians [4]. Its international promulgation improved patient outcomes and provider resuscitation skills [5–7]. Courses such as ATLS, pediatric advanced life support (PALS), and advanced cardiac life support (ACLS) advocate a standardized and systematic approach to assessment and evaluation of patients [8, 9]. The principles that providers learn from these courses enhance the delivery of care, however, retention of information does decrease as time from the last course increases [10].

In acute emergencies, the adopted standardized approach recommends a focused history with a primary survey during resuscitation followed by a secondary survey [11]. This model is based on teachings from courses such as ATLS and PALS [4, 9].

Checklists have improved systematic approaches in emergencies, trauma and intubations [12–16]. Checklists supplement the clinician’s approach by reducing omissions and improving adherence to protocol [13, 14]. Advances in medical informatics, human factors engineering and an emerging use of checklists contributed to the development of ‘Checklist for Early Recognition and Treatment of Acute Illness and Injury (CERTAIN)’ [15]. CERTAIN is a web based clinical decision support (CDS) tool that aims to combine relevant clinical information with evidence based knowledge and best clinical practices. The software is designed to assist practitioners with point of care decision support when presented with acutely decompensating patients, by providing the ability to track patient information and resuscitation actions along with providing reference tools for common resuscitation scenarios [15, 17, 18]. It is designed to have two modules, a stabilization module entitled evaluation of life threatening emergencies (ELITE) and a rounding module.

**Fig. 1:**
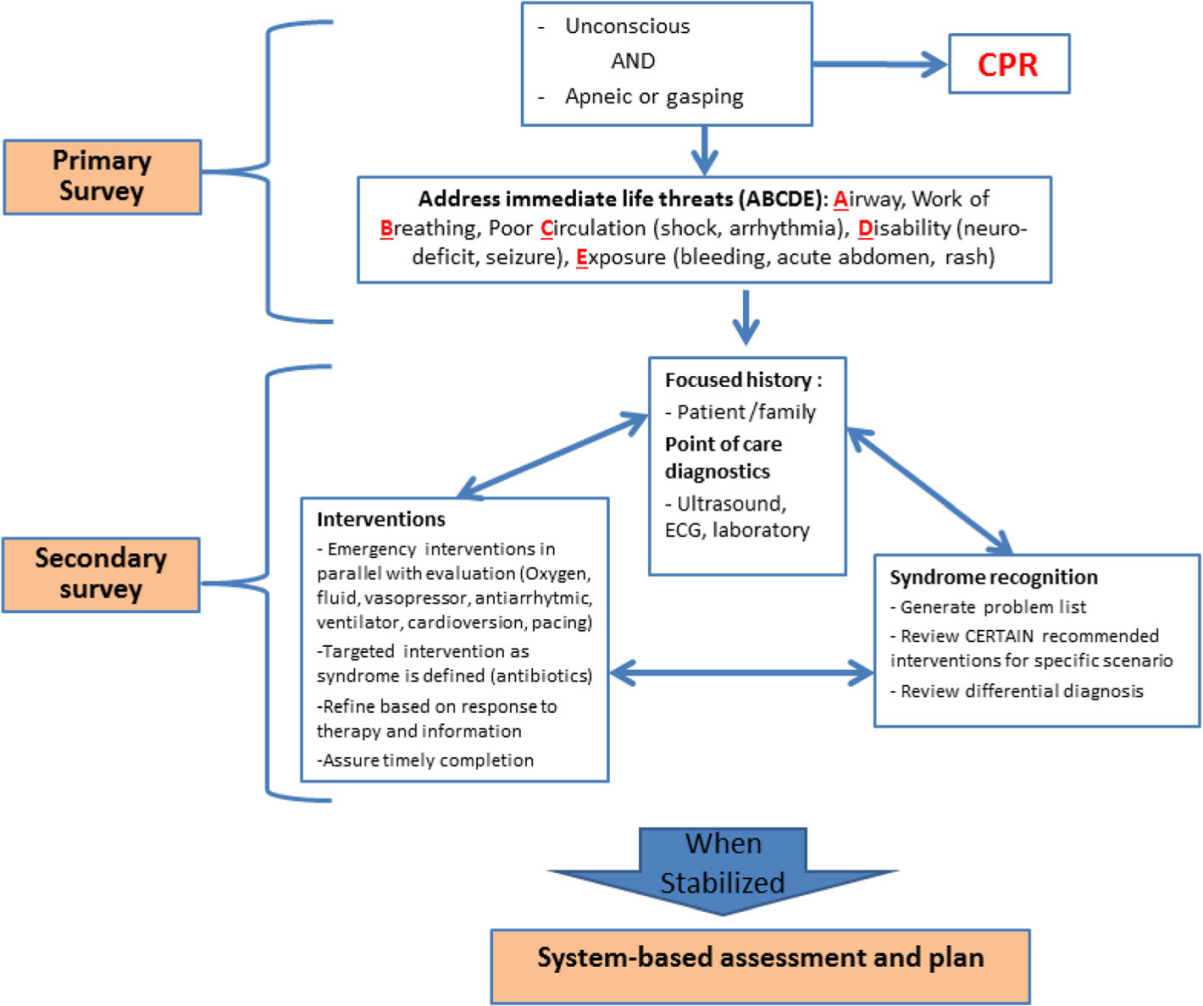
Model for approach in evaluation of critically ill patients

In designing CERTAIN’s stabilization module data elements were included that were based on mental decision making models utilized by experts in resuscitation [15, 17]. This approach (Fig. 1), much like what is used in ATLS and PALS, uses a primary and secondary survey with a focused history [15]. In CERTAIN once the reason for admission is recognized, elements used for the primary survey include airway (A): airway compromise, stridor, wheezing; breathing (B): poor air entry, crackles, work of breathing; circulation (C): ECG monitor, weak pulse, mottling; disability (D): responsiveness on an AVPU scale, weak pulse, mottling; exposure (E): abdominal distension, bleeding, skin findings. In the adult non-trauma critically ill patient, the focused history requires elements that differ from the approach adopted in trauma, cardiac arrest or assessments of pediatric patients. ATLS and PALS recommend the use of the acronym ‘SAMPLE’ for obtaining the focused history [4, 9]. Symptoms (S), allergies (A), medications (M), past medical history (P), last oral intake (L), events (E) surrounding the presenting complaint are obtained in a systematic approach to establishing critical components of the history. In the adult non-trauma patient, however, additional elements such as the specific class of medications, co-morbidities and code status can provide valuable information. In developing CERTAIN, expert recommendations were used to identify which classes of drugs were important to highlight in the assessment of life threatening illness and similarly which co-morbidities.

**Fig. 2:**
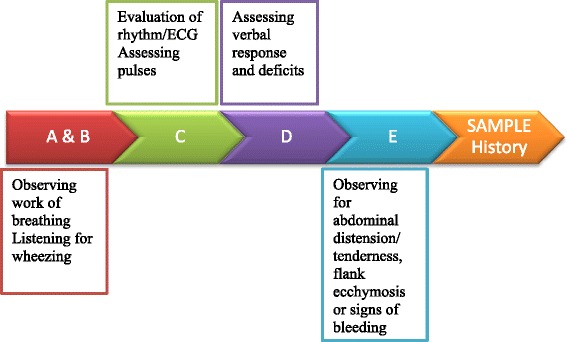
Series choreography

**Fig. 3:**
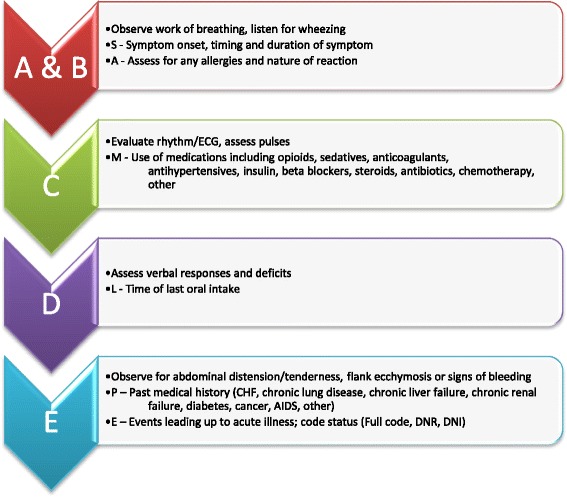
Parallel choreography

Despite the identification of key elements of a focused history in the evaluation of the critically ill adult non-trauma patient, the best way to choreograph this step into the initial assessment and stabilization phase is yet to be determined. Two possible choreographies include performing the focused history in series (Fig. 2), after, the primary survey or in parallel (Fig. 3) to the primary survey.

The hypothesis of this pilot study was that in a simulation involving an acutely ill patient, a critical care clinician would find a difference in the feasibility to perform a focused history in series to the clinical assessment as compared to in parallel to the clinical assessment.

## Methods

This was a simulation based observational study designed to compare the feasibility of choreographing a focused SAMPLE history for non-trauma critically ill patients, in series to the clinical examination and assessment compared to in parallel. The study was approved by the Mayo Clinic Institution Review Board with IRB application # 12-007998 and requirement for written informed consent was waived.

The following elements were combined for the SAMPLE history in the evaluation of the non-trauma patient by combining the principles of ATLS, PALS and expert consensus identified at the time of development of CERTAIN.

- **S** – Symptom onset, timing and duration
- **A** – Allergies and nature of reaction
- **M** – Medications (opioids, sedatives, anticoagulants, anti-hypertensives, insulin, beta blockers, steroids, antibiotics, chemotherapy, other)
- **P** – Past medical history (CHF, chronic lung disease, chronic liver failure, chronic renal failure, diabetes, cancer, AIDS, other)
- **L** – Time of last oral intake
- **E** – Events leading up to acute illness; code status [Full code, do not resuscitate (DNR), do not intubate (DNI)]

### Subjects

Volunteer critical care clinicians (fellows and attendings) were recruited that were at least ACLS certified and may have been ATLS and/or PALS certified. Fellows were either in their first or second year of critical care training with previous training in anesthesia, internal medicine including pulmonary medicine or emergency medicine. All fellows had previous exposure to simulation training that at a minimum included a 2 week orientation at the start of their critical care fellowships. The attendings were all board certified in pulmonary and critical care.

### Study design

**Fig. 4:**
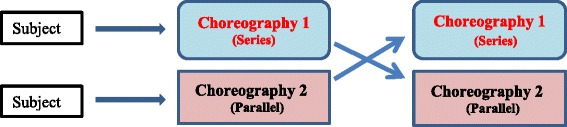
Crossover for subjects

This was an un-blinded crossover study in which volunteer consenting clinicians were randomly assigned to start with either choreography of the focused history in series or in parallel to the clinical assessment of an acutely decompensating patient. Each clinician was asked to evaluate simulation patients using a primary survey, which mimicked the primary survey components of the CDS CERTAIN, and focused history only. No patients or mannequins were used for examination during the simulation, however if the clinician asked a question regarding the examination they were provided with the finding. Following an initial choreography in series or in parallel the clinician would then crossover to complete the alternative choreography (Fig. 4).

Clinicians were given a total of six pre-determined clinical scenarios to perform, three in series and three in parallel. The cases were grouped by syndrome into hypotension (case type 1), sepsis (case type 2), and acute cardiac events (case type 3). Each clinician performed two cases from each group of syndromes, one in each of the choreographies. Prior to the start of the simulation, clinicians were informed they would be provided with a series of simulation cases in which the goal was to assess the patient through a primary survey and focused history. The determination of the diagnosis was not a required element as the focus of the study was to assess the choreography of the focused history. Participating clinicians started in either series or parallel choreography. Prompt cards that listed the A, B, C, D, and E elements of CERTAIN’s primary survey and relevant components of the SAMPLE focused history were placed in front of each participant to guide the sequence of events for the choreographies. This method was used in an attempt to mimic the use of the CERTAIN software and suggested prompter when using the ELITE stabilization module.

During each scenario the participants were timed using a stopwatch. Time recording was started when the clinicians indicated that they were ready and the case scenario began with a brief description of the presenting complaint. The total time to task completion was recorded for each of the scenarios. The tasks ended when the ‘E’ component of the SAMPLE history was fully obtained in both choreographies.

### Outcome variables

The primary outcome of this study was to determine the overall workload for series compared to parallel choreography. The NASA task load index (NASA TLX) was used for measurement of workload. NASA TLX is a rating scale that uses six component scales or domains. This integrated measure of overall workload is determined by an average of the six scales, weighted to reflect the contributions of each factor to the workload from the perspective of the rater themselves [19]. The six domains that compose NASA TLX are representations of clustered independent variables that include: mental demand, physical demand, temporal demand, performance, effort and frustration. It is assumed that the combination of these dimensions likely represent workload [20]. In this study, the paper version of NASA TLX was used. The clinician was asked to initially rate the relative importance of each of the domains. There are 15 possible pairwise comparisons for each of the six scale elements. A tally is then made of the number of times each element was selected (0 – not relevant; 5 – most relevant). The clinicians also rated the relative importance of each of the domains for completion of the task using a visual analog scale. The numeric ratings of each scale element reflect the magnitude of the factor in a given task. The final weighted task load rating for each element is the weight (tally) for that element multiplied by the magnitude of the load. The overall task load for a particular task is the sum of all the weighted task load ratings for the particular task divided by 15 [19].

The secondary outcome of this study was to determine the time to task completion, which in this case was to obtain a focused history and complete a primary survey.

### Data collection and statistical analysis

The effect of choreography was assessed using a mixed effect model. Assuming an alpha = 0.05, a mean overall work load for NASA TLX of 39 with a standard deviation of 3.0 and a constant correlation of 0.7, the estimated power was over 80 % for ten providers with three scenarios. Supposing a standard deviation of 2.0 instead of 3.0, six clinicians would have been needed to achieve 80 % power and if the standard deviation was 4.0, 14 clinicians would have been needed.

Following voluntary consent to participation, each clinician performed the clinical assessment in either series or parallel and then crossed over to the alternate choreography. Each clinician was given three scenarios to complete in each of the choreographies and was measured for scenario and choreography combinations (six NASA TLX task load surveys for each participant). A mixed effect model was used to assess the change in NASA TLX overall task load due to changes in choreography. A similar technique was used to assess differences in the other NASA TLX domain ratings. In each scenario, the median (IQR) is reported for the overall task load, the domains and the time to task completion.

## Results

Fourteen critical care clinicians (11 fellows, three attendings) volunteered for study participation. They each completed six scenarios (three in parallel and three in series) totaling 84 observations. The median (IQR) overall NASA TLX for series was 39 (17 – 58) and for parallel 43 (27 – 52), *p* = 0.57 (Additional file 1: Figure S1). Secondarily, the median (IQR) time to completion of the tasks in series was 125 (112 – 158) seconds and in parallel 122 (108 – 158) seconds, *p* = 0.92 (Additional file 1: Figure S2).

The median (IQR) for each of the domains of the NASA TLX are listed in Table 1.

**Table 1:** NASA TLX subscales

NASA TLX sub scale	Median (IQR) for Series	Median (IQR) for Parallel	Wilcoxon Sign Test, *P* =
Mental demand	30 (15 – 65)	35 (25 – 70)	**0.03**
Physical demand	10 (5 – 30)	10 (5 – 31.25)	0.48
Temporal demand	32.5 (15 – 61.25)	40 (25 – 56.25)	0.10
Performance	30 (15 – 70)	30 (20 – 55)	0.14
Effort	27.5 (10 – 40)	30 (18.75 – 46.25)	**0.03**
Frustration	20 (8.75 – 41.25)	35 (20 – 51.25)	0.06

Data in bold reflect statistical significance, alpha is set at *p* = 0.05

Parallel choreography was associated with a statistically significant but not clinically significant difference in mental demand and effort domains. NASA TLX domains for each clinician are provided in Additional file 1: Table S1.

As clinicians performed the tasks repeatedly from cases 1 to 3, the median overall NASA TLX and time to task completion improved for both series and parallel (Additional file 1: Figures S3–S5). In both choreographies, as clinicians moved from cases 1 to 3 the median overall NASA TLX improved. Median (IQR) overall NASA TLX for case 1 in series was 45 (20 – 59) and in parallel 46 (38 – 53) and for case 3 the median (IQR) in series was 35 (12 – 58) and in parallel 38 (22 – 52), *p* = 0.002. Median (IQR) time to completion for case 1 in series was 140 (120 – 178) seconds and in parallel 142 (117 – 164) seconds and for case 3 median (IQR) for series was 117 (99 – 138) seconds and for parallel was 116 (106 – 137) seconds, *p* <0.001.

During an informal debriefing, most clinicians generally stated a preference for a parallel model (Additional file 1: Table S2).

## Discussion

This simulation study found that the incorporation of a focused history into the initial assessment and evaluation of the adult non-trauma critically ill patient is feasible. There was no difference in the cognitive load and time to completion between series or parallel choreographies. However, there was a subjective preference amongst the sample providers for the parallel choreography. This was not formally measured or evaluated and differed from the results found in the study which showed a higher median NASA TLX for the domains of mental demand and effort in parallel compared to series choreography. Whether performed in series or parallel, with practice and repetition of the task, the cognitive load decreased and the time to task completion improved.

The assessment of patients with critical illness includes a focused history with evaluation of physical signs and symptoms that help prompt a diagnosis and syndrome recognition [11]. Combining principles of ATLS, ACLS, PALS and expert consensus on approaching the assessment and evaluation of the critically ill patient, essential elements of a focused history were selected [4, 8, 9, 15, 21]. These elements were then organized based on the acronym ‘SAMPLE’ and targeted for use in the simulated evaluation of an acutely decompensating critically ill patient. During the simulation, pre – defined case scenarios were used and participating clinicians were given prompt cards that listed the components of the focused history. Prompt cards specified what each of the letters of the ‘SAMPLE’ acronym represented and also listed the components of past medical history and medications that were considered vital as per expert consensus [15, 21]. Prompt cards also highlighted the ABCDE elements of the primary survey available in CERTAIN. The use of prompt cards removed the need for providers to memorize what the ‘SAMPLE’ acronym stood for or the sequence of events required for each choreography. This approach of prompting is recommended when using the CDS CERTAIN to help in the assessment and resuscitation of a critically ill patient. In a previous simulation study the use of a prompter that pointed out key elements in CERTAIN reduced omission rates [15]. Use of CERTAIN as a CDS tool has the potential to fulfill a recommendation by the Institute of medicine that health information technology be used to reduce diagnostic error [1].

The purpose of this study was to evaluate the feasibility of standardizing the approach to obtaining a focused history during the evaluation of the critically ill patient and determining the optimal choreography. Our results indicate that there was no difference in task load whether this focused history is obtained in series or in parallel. This is of importance for the incorporation of the step in any clinical decision support tool.

### Limitations

This study was limited by its simulation environment in which pre – defined scenarios were used. This does not allow for the accounting of real time variations in patients and their presentations. However, it does provide for a controlled setting for testing and comparing choreographies used in this study. Another major limitation of this study was that the providers were not tested for errors or omission. While the elements of the CDS CERTAIN (namely the primary survey components A, B, C, D, and E and the components that were identified as key elements of the history) were used, the tool itself was not used for the purposes of the simulation. The incorporation of the tool may reduce omissions in obtaining a pertinent history when assessing the critically ill patient, given that it has already been shown to reduce errors and omission rates, but this was not tested [15]. Further, while participants in the study were informally debriefed at the end of their case simulations, there was no formal evaluation of perceptions. This limits the ability to evaluate the discrepancies identified between the indicated preference of parallel choreography despite increased NASA TLX scores for mental demand and effort domains.

## Conclusions

In this pilot simulation study, we conclude that a focused history can be done in either series or parallel to the primary survey, however, there is less mental demand and effort associated with a parallel choreography. Training and practice improves both cognitive burden and time for task completion. Reducing cognitive burden and time to task completion during the initial evaluation of the acutely decompensating patient through the primary survey and a focused history may have the potential to improve outcomes. However, further evaluation incorporating assessments beyond the primary evaluation are required to determine the impact on outcomes.

## Additional file

Additional file 1: Figure S1. Overall NASA TLX scores series versus parallel. **Figure S2**, Time to task completion series versus parallel. **Figure S3**, Series choreography NASA TLX per type of case per subject. **Figure S4**, Parallel choreography NASA TLX per type of case per subject. **Figure S5**, Time to task completion per case type. **Table S1**, NASA TLX subscales per subject for series and parallel. **Table S2**, Comments from participants during debriefing. (DOCX 130 kb)

## Abbreviations

ACLS: Advanced cardiac life support
APLS: Advance pediatric life support
ATLS: Advanced trauma life support
CDS: Clinical decision support
CERTAIN: Checklist for early recognition and treatment of acute illness and injury
ELITE: Evaluation of life threatening emergencies
NASA TLX: NASA task load index
SAMPLE: acronym for symptoms (S), allergies (A), medications (M), past medical history (P), last oral intake (L), events (E) surrounding the presenting complaint
