# Supplementary material for: The incorporation of focused history in checklist for early recognition and treatment of acute illness and injury
Source: BMC Emerg Med. 2016 Aug 31;16(1):35. doi: 10.1186/s12873-016-0099-9 (PMC5006415; doi:10.1186/s12873-016-0099-9)
Supplement: Additional file 1: Figure S1. — Overall NASA TLX scores series versus parallel. Figure S2, Time to task completion series versus parallel. Figure S3, Series choreography NASA TLX per type of case per subject. Figure S4, Parallel choreography NASA TLX per type of case per subject. Figure S5, Time to task completion per case type. Table S1, NASA TLX subscales per subject for series and parallel. Table S2, Comments from participants during debriefing. (DOCX 130 kb) [file 12873_2016_99_MOESM1_ESM.docx]

# Additional file 1

**Figure e1**: Overall NASA TLX

Series choreography Parallel choreography


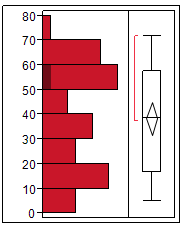

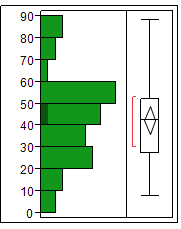


**Figure e2**: Time to task completion

Series choreography Parallel choreography


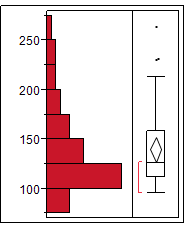

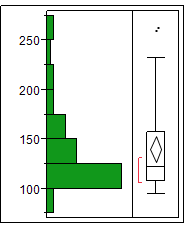


**Figure e3**: Series choreography NASA TLX per type of case per subject


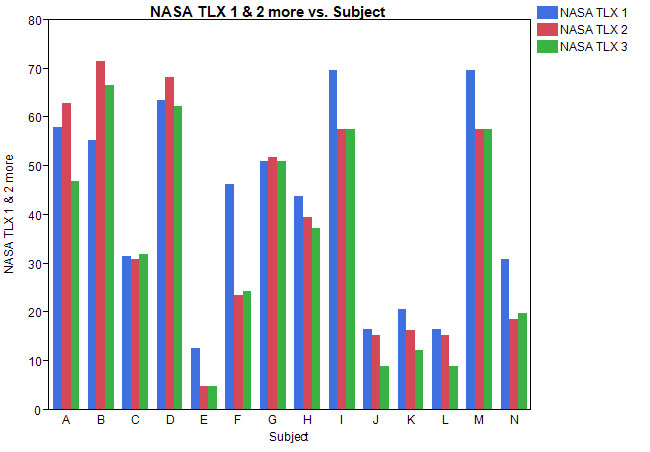


**NASA TLX Case 1 NASA TLX Case 2 NASA TLX Case 3**

**Median (IQR)**

| 45 (20 – 59) | 35 (16 – 59) | 35 (12 – 58) |
| --- | --- | --- |

**Figure e4**: Parallel choreography NASA TLX per type of case per subject


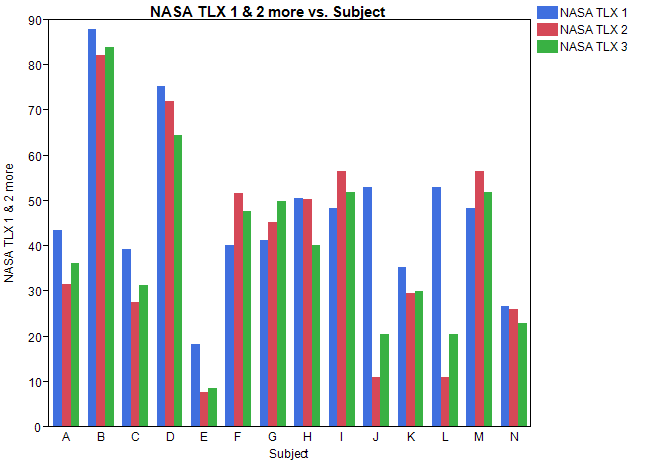


**NASA TLX Case 1 NASA TLX Case 2 NASA TLX Case 3**

**Median (IQR)**

| 46 (38 – 53) | 39 (22 – 57) | 38 (22 – 52) |
| --- | --- | --- |

**Figure e5:** Time to completion of task per case type

**Time case 1 Time case 2 Time case 3**

**Table E1**: NASA TLX subscales per subject for series and parallel

| Subject | NASA TLX subscale | Mean raw rating series  (+ SD) | Mean raw rating parallel (+ SD) | Wilcoxon Signed Rank, P = |
| --- | --- | --- | --- | --- |
| A | Mental demand | 68.3 (+ 2.9) | 35 (+ 8.7) | 0.25 |
|  | Physical demand | 60 (+ 8.6) | 35 (+ 5) | 0.25 |
|  | Temporal demand | 71.7 (+ 5.8) | 43.3 (+ 5.8) | 0.25 |
|  | Performance | 40 (+ 17.3) | 33.3 (+ 10.4) | 0.5 |
|  | Effort | 60 (+8.7) | 43.3 (+ 10.4) | 0.25 |
|  | Frustration | 61.7 (+ 2.9) | 33.3 (+ 2.9) | 0.25 |
| B | Mental demand | 73.3 (+ 20.8) | 88.3 (+ 2.9) | 0.5 |
|  | Physical demand | 15 (+ 5) | 18.3 (+ 14.4) | 1.0 |
|  | Temporal demand | 33.3 (+ 18.9) | 51.7 (+ 10.4) | 0.25 |
|  | Performance | 90 (+ 0) | 83.3 (+ 5.8) | 0.5 |
|  | Effort | 48.3 (+ 12.6) | 76.7 (+ 23.1) | 0.5 |
|  | Frustration | 31.7 (+ 30.6) | 91.7 (+ 2.9) | 0.25 |
| C | Mental demand | 18.3 (+ 2.9) | 23.3 (+ 10.4) | 0.75 |
|  | Physical demand | 8.3 (+ 2.9) | 11.7 (+ 2.9) | 0.5 |
|  | Temporal demand | 21.7 (+ 7.6) | 31.7 (+ 7.6) | 0.25 |
|  | Performance | 46.7 (+ 20.2) | 43.3 (+ 2.9) | 0.75 |
|  | Effort | 16.7 (+ 5.8) | 30 (+ 5) | 0.25 |
|  | Frustration | 41.7 (+ 7.6) | 40 (+ 5) | 1.0 |
| D | Mental demand | 58.3 (+ 5.8) | 75 (+ 5) | 0.25 |
|  | Physical demand | 58.3 (+ 5.8) | 56.7 (+ 2.9) | 1.0 |
|  | Temporal demand | 58.3 (+ 5.8) | 71.7 (+ 10.4) | 0.25 |
|  | Performance | 88.3 (+ 2.9) | 55 (+ 5) | 0.25 |
|  | Effort | 38.3 (+ 2.9) | 78.3 (+ 7.6) | 0.25 |
|  | Frustration | 31.7 (+ 10.4) | 61.7 (+ 11.5) | 0.025 |
| E | Mental demand | 5 (+ 0) | 13.3 (+ 10.4) | 0.5 |
|  | Physical demand | 5 (+ 0) | 16.7 (+ 16.1) | 0.5 |
|  | Temporal demand | 5 (+ 0) | 8.3 (+ 2.9) | 0.5 |
|  | Performance | 10 (+ 8.7) | 6.7 (+ 2.9) | 1.0 |
|  | Effort | 8.3 (+ 5.8) | 11.7 (+ 5.8) | 1.0 |
|  | Frustration | 5 (+ 0) | 13.3 (+ 10.4) | 0.5 |
| F | Mental demand | 30 (+ 10) | 66.7 (+ 20.2) | 0.25 |
|  | Physical demand | 10 (+ 0) | 8.3 (+ 2.9) | 1.0 |
|  | Temporal demand | 33.3 (+ 10.4) | 40 (+ 15) | 1.0 |
|  | Performance | 35 (+ 34.7) | 20 (+ 8.7) | 1.0 |
|  | Effort | 33.3 (+ 15.3) | 36.7 (+ 15.3) | 1.0 |
|  | Frustration | 13.3 (+ 7.6) | 51.7 (+ 7.6) | 0.25 |
| G | Mental demand | 28.3 (+ 5.8) | 28.3 (+ 5.8) | 1.0 |
|  | Physical demand | 5 (+ 0) | 5 (+ 0) | 1.0 |
|  | Temporal demand | 65 (+ 5) | 58.3 (+ 7.6) | 0.5 |
|  | Performance | 45 (+ 5) | 36.7 (+ 2.9) | 0.25 |
|  | Effort | 30 (+ 13.2) | 30 (+ 5) | 1.0 |
|  | Frustration | 63.3 (+ 2.9) | 58.3 (+ 2.9) | 0.5 |
| H | Mental demand | 68.3 (+ 5.8) | 76.7 (+ 5.8) | 0.25 |
|  | Physical demand | 5 (+ 0) | 5 (+ 0) | 1.0 |
|  | Temporal demand | 50 (+ 0) | 60 (+ 8.7) | 0.25 |
|  | Performance | 30 (+ 13.2) | 30 (+ 13.2) | 1.0 |
|  | Effort | 5 (+ 0) | 5 (+ 0) | 1.0 |
|  | Frustration | 5 (+ 0) | 5 (+ 0) | 1.0 |
| I | Mental demand | 66.7 (+ 7.6) | 53.3 (+ 10.4) | 0.5 |
|  | Physical demand | 40 (+ 8.7) | 35 (+ 8.7) | 0.25 |
|  | Temporal demand | 61.7 (+ 12.6) | 50 (+ 8.7) | 0.75 |
|  | Performance | 65 (+ 8.7) | 60 (+ 5) | 0.5 |
|  | Effort | 56.7 (+ 12.6) | 53.3 (+ 7.6) | 1.0 |
|  | Frustration | 50 (+ 21.8) | 35 (+ 0) | 0.5 |
| J | Mental demand | 13.3 (+ 7.6) | 36.7 (+ 34) | 0.5 |
|  | Physical demand | 5 (+ 0) | 5 (+ 0) | 1.0 |
|  | Temporal demand | 13.3 (+ 2.9) | 30 (+ 30.4) | 0.75 |
|  | Performance | 13.3 (+ 2.9) | 20 (+ 10) | 0.5 |
|  | Effort | 16.7 (+ 7.6) | 21.7 (+ 5.8) | 1.0 |
|  | Frustration | 6.7 (+ 2.9) | 13.3 (+ 10.4) | 0.5 |
| K | Mental demand | 20 (+ 8.7) | 38.3 (+ 2.9) | 0.25 |
|  | Physical demand | 8.3 (+ 2.9) | 10 (+ 0) | 0.5 |
|  | Temporal demand | 15 (+ 5) | 31.7 (+ 7.6) | 0.25 |
|  | Performance | 15 (+ 0) | 23.3 (+ 2.9) | 0.25 |
|  | Effort | 20 (+ 10) | 35 (+ 5) | 0.25 |
|  | Frustration | 13.3 (+ 5.8) | 30 (+ 0) | 0.25 |
| L | Mental demand | 5 (+ 0) | 5 (+ 0) | 1.0 |
|  | Physical demand | 5 (+ 0) | 5 (+ 0) | 1.0 |
|  | Temporal demand | 5 (+ 0 ) | 5 (+ 0) | 1.0 |
|  | Performance | 13.3 (+ 2.9) | 11.7 (+ 2.9) | 1.0 |
|  | Effort | 6.7 (+ 2.9) | 5 (+ 0) | 1.0 |
|  | Frustration | 18.3 (+ 2.9) | 20 (+ 0) | 1.0 |
| M | Mental demand | 36.7 (+ 5.8) | 36.7 (+ 11.5) | 1.0 |
|  | Physical demand | 31.7 (+ 2.9) | 33.3 (+ 5.8) | 1.0 |
|  | Temporal demand | 70 (+ 0) | 70 (+ 18.0) | 1.0 |
|  | Performance | 70 (+ 0) | 70 (+ 8.7) | 1.0 |
|  | Effort | 33.3 (+ 2.9) | 35 (+ 10) | 1.0 |
|  | Frustration | 40 (+ 5) | 35 (+ 5) | 0.5 |
| N | Mental demand | 23.3 (+ 5.8) | 28.3 (+ 7.6) | 0.5 |
|  | Physical demand | 10 (+ 0) | 10 (+ 0) | 1.0 |
|  | Temporal demand | 25 (+ 8.7) | 28.3 (+ 2.9) | 0.75 |
|  | Performance | 21.7 (+ 2.9) | 23.3 (+ 2.9) | 1.0 |
|  | Effort | 23.3 (+ 10.4) | 21.7 (+ 2.9) | 1.0 |
|  | Frustration | 25 (+ 8.7) | 26.7 (+ 7.6) | 1.0 |

**Table E2**: Comments from participants during debriefing

| Artificial environment, no preference |
| --- |
| In simulation scenarios I liked series better. Parallel was difficult because of prompts. |
| Preference: Ideally should be parallel in the ICU. Series may be well suited to outpatient medicine. Further studies should focus on real time simulation in sim lab. |
| Series is better for critically ill patients, less demand mentally. SAMPLE organizes afterward. |
| After I completed the study, I realized that I like the parallel model of getting the history. |
| Prefer parallel over series. |
| Parallel seemed to provide more clinically useful information in a more rapid fashion. Likely less time needed with parallel compared to stepwise series investigation/history. |
| I feel that the series approach is more similar to my current practice and therefore easier for me to accomplish. |
